# Cardiovascular, Pulmonary, and Neuropsychiatric Short- and Long-Term Complications of COVID-19

**DOI:** 10.3390/cells11233882

**Published:** 2022-12-01

**Authors:** Małgorzata Kobusiak-Prokopowicz, Katarzyna Fułek, Michał Fułek, Konrad Kaaz, Andrzej Mysiak, Donata Kurpas, Jan Aleksander Beszłej, Anna Brzecka, Jerzy Leszek

**Affiliations:** 1 Department of Cardiology, Institute of Heart Diseases, Wroclaw Medical University, 50-556 Wroclaw, Poland; 2 Lower Silesian Oncology, Pulmonology and Hematology Center, 53-413 Wroclaw, Poland; 3 Department and Clinic of Internal Medicine, Occupational Diseases, Hypertension and Clinical Oncology, Wroclaw Medical University, 50-556 Wroclaw, Poland; 4 Department and Clinic of Family Medicine, Wroclaw Medical University, 51-141 Wroclaw, Poland; 5 Department and Clinic of Psychiatry, Wroclaw Medical University, 50-367 Wroclaw, Poland; 6 Department of Pulmonology and Lung Oncology, Wroclaw Medical University, 53-439 Wroclaw, Poland

**Keywords:** COVID-19, complications, SARS-CoV-2, cardiovascular, infection, inflammation, neuropsychiatric, pulmonary

## Abstract

Beginning with the various strategies of the SARS-CoV-2 virus to invade our bodies and manifest infection, and ending with the recent long COVID, we are witnessing the evolving course of the disease in addition to the pandemic. Given the partially controlled course of the COVID-19 pandemic, the greatest challenge currently lies in managing the short- and long-term complications of COVID-19. We have assembled current knowledge of the broad spectrum of cardiovascular, pulmonary, and neuropsychiatric sequelae following SARS-CoV-2 infection to understand how these clinical manifestations collectively lead to a severe form of the disease. The ultimate goal would be to better understand these complications and find ways to prevent clinical deterioration.

## 1. Introduction

Since the initial outbreak in December 2019 in Wuhan, China, the scale and character of the COVID-19 pandemic have continued to change and continue to reveal the unique and evolving pathogenicity of the SARS-CoV-2 virus. To date, more than 370 million people have contracted COVID-19, and more than 5.5 million people have died from this disease [1].

Although we do not have sufficient information to know the detailed impact of this disease, it is becoming increasingly evident that SARS-CoV-2 infection is responsible for short- and long-term complications. To contain mortality during the initial phase of the pandemic, scientific attention has been initially directed toward studying the acute nature of the disease. Over time, the association between SARS-CoV-2 infection and short- as well as long-term pulmonary, cardiovascular, and neuropsychiatric complications became apparent [2,3,4]. There is marked heterogeneity in the short and long-term complications seen following COVID-19 illness, including hematological [5], nephrological [6], and endocrinological complications [7], which are not included in this review.

COVID-19 can manifest clinically in a mild to critical form, with most patients developing only the milder or asymptomatic form [8]. In approximately 5% of patients, the severe form of COVID-19 occurs with septic shock, acute respiratory distress syndrome (ARDS), thromboembolic events, multi-organ failure, acute renal, and cardiac damage [9]. In addition, some patients with COVID-19 fail to fully recover from the disease and suffer a range of complications affecting multiple organs in the form of post-COVID-19 syndrome or long COVID [10].

In this review, we have synthesized what is known about the cardiovascular, pulmonary, and neuropsychiatric complications of COVID-19 and discuss how they interact to manifest the critical nature of the disease.

## 2. Relationship between COVID-19 and Cardiovascular Disorders

SARS-CoV-2 infection can lead to severe but probably reversible cardiovascular dysfunction without clinically significant pulmonary manifestations. The pathophysiologic causes of myocardial dysfunction in patients with COVID-19 are not yet clear. However, several pathobiological mechanisms are suspected: stress cardiomyopathy, macrovascular or microvascular supply–demand mismatch, cytokine storm, and myocarditis with or without pericarditis [11,12]. Most of them can lead to neurological complications.

Stress cardiomyopathy is a syndrome characterized by chest pain, ECG changes (ST-segment), elevated serum troponin levels, and echocardiographic changes (myocardial dysfunction) [13]. It classically occurs in older women, typically after emotional or physical stress. An intracranial hemorrhage or electroconvulsive therapy can cause cardiomyopathy and frequently develops T-wave inversion and QT interval prolongation. Based on this circumstance and the phenotypic overlap of this syndrome with cardiomyopathy, a neurocardiogenic mechanism has been suggested [14].

Elevated serum levels of troponin are associated with an increased risk of arrhythmias, hypoxemic respiratory failure, and death in hospitalized patients with COVID-19. Stress and infection can trigger plaque rupture in epicardial coronary arteries leading to myocardial infarction [2,3,15,16].

In patients with COVID-19, elevated D-dimer levels have been associated with severe disease, often with multisystemic disorders. The microvascular thrombosis due to a thromboinflammatory state may be common in the patients with disseminated intravascular coagulation, thrombocytopenia, and a consumptive coagulopathy. The increase in activated partial thromboplastin time that occurs in some cases has been suggested to be due to a lupus anticoagulant [17,18]. In some cases, microthrombi have been reported in the liver, kidney, or lung, but microthrombi in the heart has not been described in reports of patients with severe myocardial dysfunction or in reports of postmortem examinations of patients with COVID-19. Microthrombi that is widespread enough to cause the degree of myocardial dysfunction results in persistent elevation of cardiac troponin T levels. Endotheliitis, reported in various tissues, may result from direct penetration of the virus or from cytokine-mediated effects on the endothelium, possibly altering its permeability and leading to myocardial edema [19,20].

A jump in inflammatory cytokines has been observed in patients receiving T-cell therapy with chimeric antigen receptors (CAR). This likely plays a role in the multisystemic dysfunction that can occur in patients with severe COVID-19 and is characterized by very high levels of C-reactive protein, interleukin-6, and ferritin [21].

The co-occurrence of chronic diseases in the elderly is a common problem, but the relationship between comorbidities and the prognosis of elderly patients with COVID-19 is unclear [22].

Dai et al. divided the study patient population into an elderly group (≥60 years old) and a nonelderly group (<60 years old). The proportion of severe cases was higher in the elderly group than in the nonelderly group (73.9% versus 42.2%). Older patients with COVID-19 had a relatively higher proportion of comorbidities, and the most common were atherosclerotic cardiovascular disease (56.5%) and hypertension (43.5%) [22]. However, no direct association was found between hypertension and COVID-19 in elderly patients. In an observational cohort study by Trecarichi et al., 50 patients with a mean age of 80 years were included. In a cohort of elderly patients with COVID-19 and cardiovascular disease other than hypertension, a high in-hospital mortality rate of 32% was observed [23].

On the other hand, studies reported that hypertension may play an important role in the unfavorable outcomes of COVID-19 but still contain many confounding factors. In a meta-analysis that included 24 observational studies [24], 99,918 COVID-19 patients were included. The proportion of hypertension in critical COVID-19 patients was 37% compared with 18% of noncritical COVID-19 patients; among those who died, it was 46% compared with 22% of survivors. The results showed that patients with hypertension had a 1.82-fold higher risk of developing critical COVID-19 and a 2.17-fold higher risk of COVID-19-related mortality [24]. The results of the meta-regression analysis showed that age significantly influenced the association between hypertension and COVID-19-related mortality [24]. A comparison of blood pressure thresholds and targets between ACC/AHA, ACP/AAFP, and ESC/ESH guidelines is shown in Table 1 [25].

Hypertension is common in older adults and is often undertreated. In the very elderly admitted to a geriatric hospital for COVID-19, mortality was lower in those treated with ARB or ACEI before the onset of infection. Continuation of ACEI/ARB therapy should be encouraged in elderly patients during periods of coronavirus outbreak [26].

In contrast to adults, pediatric COVID-19 infection is asymptomatic 90% of the time. In other cases, less than 10% of children, the course of pediatric COVID-19 is reported to be a mild disease. There is still no scientific explanation for why children are affected less by COVID-19 [27]. However, a specific disease syndrome was observed in children in population studies that resembled severe Kawasaki-like disease in association with hyperinflammatory shock. It was named multisystem inflammatory syndrome in children (MIS-C) associated with COVID-19 [28]. Children may develop MIS-C after mostly an asymptomatic COVID-19 infection.

By definition, the diagnosis includes fever, laboratory evidence of inflammation, and evidence of clinically severe illness requiring hospitalization with multisystem organ involvement in a person < 21 years with COVID-19. The above diagnosis should be made after excluding the alternative diagnoses [29]. Cardiac involvement occurs in up to 67–80% of children with MIS-C and includes ventricular dysfunction, coronary artery aneurysms, conduction abnormalities, and arrhythmias. A reduction in left ventricular ejection fraction is the most commonly reported finding, occurring in 34–50% of children [29,30]. This article focuses on adults, but as MIS-C also applies to young adults, it is worth mentioning. An extensive meta-analysis by Radia et al. confirmed that cardiac involvement was evident in the majority of cases where cardiac investigations were performed and cardiovascular dysfunction was the most frequently described physiological abnormality [31]. Fortunately, while the COVID-19 pandemic is more controlled, the incidence of MIS-C cases is decreasing [32].

## 3. Neuropsychiatric Complications of SARS-CoV-2 Infection

Severe environmental stress associated with pandemics has affected the human population since the 19th century. In 1839, English physician Henry Holland claimed that influenza was “responsible for the impairment of mental functions”. Over the years, it has become clear that a combination of systemic infection, viral neurotrophy, and environmental factors can promote and even cause the development of psychiatric disorders [33]. A nationwide cohort study in Denmark showed that severe infections requiring hospitalization increased the frequency of subsequent psychiatric consultations by 84% and the need for psychotropic medication by 42%. Correspondingly, less severe infections also increased this risk by 40% and 22%, respectively [34].

The primary targets of SARS-CoV-2 are epithelial cells of the pulmonary and gastrointestinal tracts. However, the invasion of the virus is most likely not limited to these organs; it could enter the body through several pathways. The leading and most studied strategy is binding to human angiotensin-converting enzyme 2 (ACE2) as an entry receptor and human proteases as entry activators. ACE2 is expressed in many tissues, including the central nervous system [35,36].

Another postulated entry point for SARS-CoV-2 to the brain, with a little protection of the blood–brain barrier (BBB), is circumventricular organs, making it prone to various types of inflammation (including sepsis, stress, or autoimmune encephalitis).

An inflammatory storm and the massive release of inflammatory signaling factors (e.g., cytokines and chemokines) also contribute to the weakening of the BBB, which enhances the neuroinflammatory process [37].

Anosmia, which was one of the first described extrapulmonary manifestations of SARS-CoV-2, also raises the question of whether the virus is transported through the axons of olfactory bulb neurons and infects the brain via this pathway [38].

Whether SARS-CoV-2, like other coronaviruses (e.g., SARS-CoV-1 or MERS-CoV), is neurotropic requires further investigation. Other ss-coronaviruses have been found to extend their presence beyond respiratory cells and frequently affect the central nervous system [37].

Clinically, neurological and psychiatric signs or symptoms, for example headache, impaired consciousness, paresthesias, occur in 36.4% of hospitalized patients with COVID-19, with a higher incidence in patients with more severe disease [39].

Due to the clinical picture, neuropsychiatric complications of SARS-CoV-2 infection could be divided into two more distinct groups: psychiatric and neurological. Such a distinction may be useful in organizing specialist consultations and treatment procedures and may be related to the causes of the symptoms. In these two groups, symptoms may overlap and may be causally related.

### 3.1. Neurological Symptoms or Disorders

This subgroup of symptoms can also be divided into those that appear quickly, almost simultaneously with the early symptoms of systemic SARS-CoV-2 infection, the so-called acute symptoms, and symptoms that may develop slowly or appear later, but persist longer or even chronically even when the symptoms of the infection disappear. They can also be a consequence of brain damage.

The most common acute neurological symptoms include disturbance of the perception of smell and taste, and anosmia. Research is ongoing to find out the causes of its appearance in the early stages of COVID-19, as described above.

Interesting research results were presented by the authors of Douaud G et al., *Nature* (2022), that changes in the brain in patients appeared not only after severe SARS-CoV-2 infection, requiring hospitalization, but also in milder cases of the disease. These changes have been demonstrated using magnetic resonance imaging of the brain. In addition to lesions indicating tissue damage associated with the primary olfactory cortex, researchers noticed that the brains of people after SARS-CoV-2 infection showed a reduction in gray matter thickness and tissue contrast in the orbitofrontal cortex (related to emotions, reward, and decision-making) and in the parahippocampal gyrus (which plays a major role in encoding and memory retrieval). A greater reduction in overall brain size has also been shown. These were the differences from 0.2 to 2 percent in size compared with those in the control group who were not infected. Infected study participants also had poorer cognitive functions, and the negative effects described above were more pronounced in the elderly. The authors suggest that pathological changes in the brain are a sign of the degenerative spread of the disease following the immune response of the nervous system. However, it can also be influenced by the lack of sensory stimuli caused by anosmia. Yet, it is still unknown what exactly causes long COVID, or why it affects even people who have been mildly ill [40].

Except for anosmia, headache, dizziness, and impaired consciousness were the most commonly reported neurologic symptoms of COVID-19 [30]. Some patients present primarily with neurologic symptoms (e.g., stroke or epilepsy) without a typical history of respiratory distress with choking [41,42]. Such symptoms are not specific to SARS-CoV-2 infection and may occur in other viral infections and could also occur via indirect mechanisms of neuropathogenicity, e.g., as a result of respiratory distress, hypoxia, hypotension, dehydration, or fever during sepsis [42].

Delirium and memory loss observed in elderly patients recovering from pneumonia appear to be due to neuroinflammation, which has been well-described as a major component of neurodegenerative diseases [43].

In recent months, reports of meningitis, encephalitis, myelitis, or peripheral nerve damage have been published by COVID-19, suggesting that SARS-CoV-2 can directly infect structures of the nervous system. A 24-year-old adult from Japan was the first patient diagnosed with meningitis/encephalitis in March 2020 [42]. It was the first case in which SARS-CoV-2 RNA was detected in the CSF, demonstrating the neuroinvasive potential of the virus. Consequently, viral encephalitis as a complication of coronavirus infection is considered by clinicians to recognize the respiratory symptoms of encephalitic brainstem injury and as an independent disease.

In clinical practice, respiratory failure in some COVID-19 patients is manifested by decreased respiratory rate—a likely effect of the COVID-19-dependent alteration in baroreceptor function. Coronaviruses mainly affect neurons in the brainstem and damage sensitive cardio-respiratory control areas, exacerbating disease progression or even leading to respiratory failure [33].

Similar to SARS-CoV-1, SARS-CoV-2 can cause Guillain–Barre syndrome, which is characterized by weakness, difficulty walking and breathing, and difficulty weaning from artificial ventilation [41].

The increasing number of reports of COVID-19 patients with neurological problems and experimental models demonstrating neuroinvasion raise concerns that SARS-CoV-2 is a novel neuropathogen that remains underdiagnosed [44]. During the COVID-19 pandemic, it is also important to provide continuous care for people with chronic neurologic disorders.

In addition to neuroinvasion, COVID-19 infection triggered immunity and caused massive inflammation of the lungs and brain; the former is the main cause of death in COVID-19, and the latter can lead to brain hemorrhage. Hypercoagulation and aneurysm instability, partly due to systemic COVID-19 inflammation, may be the possible cause manifestation of neurological symptoms.

The neurological manifestations are currently supported by the following mechanisms, previously described in agreement with Heneka et al. (2020): (1) direct viral encephalitis, (2) systemic inflammation, (3) peripheral organ dysfunction (liver, kidney, lung), and (4) cerebrovascular changes. However, in most cases, the neurological manifestations of COVID-19 may result from a combination of the above factors [45,46]. Panariello et al. (2020) proposed a fifth possible mechanism related to the pathogenesis of SARS-CoV-2 infection or the binding of the virus to ACE2, leading to a downregulation of this receptor and a change in the dynamic balance between the two arms of the RAAS: (1) ACE/Ang II/AT1R with proinflammatory activity and (2) ACE2/Ang-(1-7)/MasR with anti-inflammatory properties [46].

### 3.2. Psychiatric Symptoms or Disorders

As previously mentioned in SARS-CoV-2 infection, symptoms of a mental disorder may overlap with neurological symptoms. Immune changes in the brain during an infection can also aggravate psychiatric problems that existed before the infection.

Inflammatory disorders are also responsible for many other neuropsychiatric disorders such as schizophrenia, depression, bipolar disorder, and multiple sclerosis [47]. Cytokine levels related to disease activity remain low in patients with unaffected mood but rise sharply in patients who resist treatment [48].

In COVID-19-positive patients with severe disease progression, an exaggerated host immune response is observed. An abnormally high serum level of IL-6 detected in patients with COVID-19 is an important inflammatory biomarker and could be used to predict disease severity [49]. Alterations in IL-6 levels are also observed in psychiatric disorders such as major depression, schizophrenia, or suicide attempts.

Following the research on other coronaviruses (SARS-CoV-1 and MERS-CoV), it is very likely that some people do not recover their mental state or cognitive abilities after recovery from a physical illness such as pneumonia [50]. In addition, the number of patients with severe COVID-19 increases rapidly beyond the age of 55, as does the mortality rate [51]. Aging is an important risk factor for cognitive impairment; in addition, systemic diseases and stress may accelerate the onset of the disease. COVID-19 pandemic-related reductions in interpersonal communication with subsequent alienation, especially among primarily vulnerable groups such as the elderly or teenagers, can lead to depression and anxiety and increase suicide risk [52]. This is not only due to the ability of the virus to invade the CNS and damage neuronal cells, but also because patients face an untreatable and potentially fatal disease that can cause persistent behavioral changes or exacerbate pre-existing mental illness. The long-term consequences will only become apparent in the coming months to years.

COVID-19 is associated with neuropsychiatric complications, of which anxiety is the most common. Several biological and psychosocial factors contribute to anxiety in COVID-19. Biological factors include stress, genetics, gender, immune system, resilience, anosmia, hypogeusia, direct central nervous system (CNS) infection with SARS-CoV-2, and comorbid psychiatric and general medical conditions, ARDS, and ICU stay. Anosmia and hypogeusia are COVID-19-specific risk factors for anxiety. Knowledge of anxiety risk factors is important to focus on timely interventions, as anxiety can exacerbate COVID-19 progression. There is an inverse correlation between resilience and anxiety due to COVID-19, and efforts should be made to increase resilience in COVID-19 patients. In COVID-19, one of the main causes of anxiety is neuroinflammation resulting from immune system activation and cytokine storm. The general approach to treating anxiety during COVID-19 should take a compassionate approach, similar to trauma or disaster, and attempt to provide a sense of hope and resilience. The choice of pharmacological treatment for anxiety should focus on the stress response and its effects on the immune system [53].

During the COVID-19 pandemic, individuals exhibited certain behaviors to reduce stress and anxiety. They were more likely to search the Internet for health information on wearing face masks or washing their hands. A healthy lifestyle and psychological interventions were recommended to strengthen the immune system against COVID-19. Although interdiction appears to be effective against COVID-19, it had profound effects on the social interaction and psychological well-being of the population [54].

The binding of SARS-CoV-2 to ACE2R decreases the availability of ACE2R, leading to a reduction in the downstream mechanism of CRH and lower glucocorticoid production. Consequently, fewer glucocorticoids are available to limit excessive inflammation, leading to a sustained stress response. Environmental conditions and comorbid psychiatric illness further exacerbate this cycle. SARS-CoV-2 infection and stress contribute to excessive inflammation, which can alter neurotransmitter signaling and compromise the structural integrity of neurons through multiple mechanisms. These changes can lead to abnormal levels of dopamine, glutamate, GABA, serotonin, and norepinephrine in various brain regions, including the ventral striatum, hippocampus, amygdala, raphe nuclei, and locus coeruleus, contributing to the development of psychotic, mood, and anxiety disorders or exacerbating preexisting conditions [55].

In two patients described from the psychiatric unit for COVID-19 emergencies at the College of Florence, anosmia and hyposmia were not limited to the sensory level. In both cases, patients reported a degree of depersonalization (“loss of oral cavity”) and derealization (“change in atmosphere”) [56]. Watson et al. studied 42 cases of psychosis in COVID-19-positive patients [57]. COVID-19-related psychoses were also described by Ferrando et al. in three patients (two of whom had a psychiatric history) who presented with new-onset severe panic attacks, paranoia, and disorganized thinking, without characteristic respiratory or gastrointestinal symptoms. Patients did not express COVID-19-related nervousness. Patients were physically healthy with minimal variation in laboratory tests except for inflammatory markers [58], which may support the theory that the cytokine storm is responsible for immune-mediated neuropsychiatric symptoms.

Both SARS-CoV-2 and the previous SARS-CoV infections are associated with an increase in pro-inflammatory cytokines and chemokines (cytokine storm). At the same time, the pathogenicity of MERS-CoV is based on its IFN antagonist proteins. Ongoing research shows that many psychiatric disorders are characterized by inflammation, and their treatments have different anti-inflammatory properties. The profound psychosocial impact of SARS-CoV-2 means that patients will receive standard antidepressants and antipsychotics for these disorders [55].

Direct CNS infection combined with systemic inflammation and hypoxia in COVID-19-positive patients can cause both immediate and long-term chronic neurological and psychiatric cognitive impairment. Therefore, the development of an appropriate therapeutic strategy, rapid recognition of the patient, and rehabilitation are only possible through a multidimensional, interdisciplinary team approach.

## 4. Pulmonary Complications of COVID-19

Patients with COVID-19 have symptoms mainly pertaining to the respiratory tract, accompanied by general and other organ-related complications. Radiological studies, such as chest X-rays or CT, are routine examinations in COVID-19 patients, allowing prediction of the course of the disease [59]. Although the course of COVID-19 may be asymptomatic or with mild or moderately severe symptoms, occasionally, the illness may progress to a severe form. In some cases, pulmonary difficulties develop, either in the acute or later stages of the disease. The main pulmonary complications include pulmonary fibrosis, lung function impairment, pulmonary embolism, and pneumothorax.

### 4.1. Interstitial Pulmonary Disease and Lung Ventilatory Function Impairment

After SARS-CoV-2 infection, interstitial lung disease may develop similarly to other viral infections [60]. Interstitial lung disease after COVID-19 pneumonia may present as interstitial lung fibrosis or organizing pneumonia [4,61].

Fibrotic changes after COVID-19 develop more frequently in older patients, above 50 years of age, with initially more severe lung involvement, tachycardia, prolonged hospitalization, and treatment with mechanical ventilation [62]. The other postulated risk factors for post-COVID lung fibrosis include smoking and alcoholism [63], and more intense systemic inflammation as indicated by a decreased number of platelets and leukocytes and decreased hemoglobin concentration at the time of COVID-19 pneumonia diagnosis [64]. However, lung fibrosis development has also been observed after mild or even asymptomatic SARS-CoV-2 infection [65].

The symptoms and signs of interstitial lung fibrosis developing as a consequence of COVID-19 pneumonia include dry cough, dyspnea on effort, bilateral crackles on auscultation, and desaturation during exercise [66]. Lung fibrosis in prolonged COVID-19 may constitute fatal complications, e.g., in patients with pre-existing emphysema [67].

Lung fibrosis could be diagnosed by radiological approaches, ventilatory function tests, or histological assessment. The radiological signs of post-COVID fibrosis may consist of nonspecific chronic interstitial lung disease with thickening of interlobular and intralobular septa and bronchial dilatation [65]. Fibrotic lesions may appear already at the beginning of COVID-19 in fibrotic bands in the peripheral lower regions of the lungs, accompanied by the areas of ground-glass opacities, typical for the early stage of the disease [68].

Pulmonary function impairment after SARS-CoV-2 infection belongs to main post-COVID sequelae [69]. Late pulmonary consequences of SARS-CoV-2 infection encompass limitation of ventilatory lung function of restrictive type, with decreased total lung capacity and diminished diffusion capacity of the lungs [65]. The latter sign may also be present without lung restriction [70] and can be regarded as the most sensitive tool in detecting post-COVID ventilatory impairment. The study performed on the COVID-19 patients 30 days after symptom onset revealed impaired diffusion capacity in more than one-fourth of them (26%) [70]. Similarly, decreased diffusion capacity was found in 26% of patients examined after six months from symptom onset and hospitalization because of severe COVID-19 [62]. Interestingly, the symptoms persisting after COVID-19 were not correlated with the results of pulmonary function tests, neither with diffusion capacity nor with a forced volume capacity of the lungs [71].

Histological examinations of the lung biopsy of the patients with the most severe COVID-19, taken during surgical biopsy or post-mortem, showed diffuse interstitial fibrosis [61]. A case report of the autopsy results revealed diffuse fibrosis with honeycomb remodeling of the lung in a patient who died of lung fibrosis two months after the elimination of SARS-CoV-2 [72].

Between two weeks and three months since SARS-CoV-2 infection, abnormalities in pulmonary function tests and radiological sequelae were present in 39–83% of the adults below 50 years of age [69]. The exact data regarding long-term fibrotic lung sequelae after COVID-19 remains scarce due to the short observation time [73]. Abnormalities in chest X-rays were observed in 4% of post-COVID patients at around 75 days after diagnosis [74]. The study of 837 patients discharged home after hospitalization due to COVID-19 pneumonia revealed that after four weeks, 4.8% of them showed interstitial lung disease patterns, mostly organizing pneumonia [4]. In the study of 90 patients who had a chest CT performed at the time of diagnosis, and eight weeks later, lung fibrosis was detected in one-fourth of them [64].

A longer follow-up period since the onset of the disease gives various data, and the occurrence of persistent pulmonary fibrotic changes after the acute phase of COVID-19 differs significantly in the studied patient populations and depends on the employed methods, i.e., chest X-ray vs. chest CT. In one study, among the patients followed up at 12 ± 8 weeks after hospitalization, most of them (88%) had either normal or considerably improved chest X-rays at this time [75]. Similarly, in the patients who were followed up after severe COVID-19 pneumonia, chest X-rays performed at 12 weeks revealed complete resolution of lung opacities in 72% of them [76]. In the patients who underwent moderate COVID-19 pneumonia, follow-up studies at 3 months showed residual pulmonary fibrotic changes by CT in 71% of patients [77]. In the survivors of ARDS caused by COVID-19, high-resolution CT after 3 months from discharge detected residual changes, such as linear bands, ground grass opacities, reticulations, consolidations, and bronchiectasis in 97.7% of the patients [78]. The main predictors of sustained radiological changes after COVID-19 appeared to be age, body mass index, fever, and high procalcitonin at hospitalization [79]. Elderly survivors who received mechanical ventilation constitute the highest group at risk of developing lung fibrosis after COVID-19 [80]. In the study of patients who underwent CT chest examination six months after symptom onset, radiological signs of fibrosis could be seen in 35% [62]–40% [81] of them.

Treatment with corticosteroids may lead to clinical and radiological rapid improvement in patients with interstitial lung disease during COVID-19. The patients surveyed after 4 weeks from hospital discharge due to COVID-19 pneumonia interstitial lung disease, primarily organizing pneumonia, responded to corticosteroid treatment, leading to a marked improvement in diffusion capacity and a noticeable increase in forced vital capacity [4].

Considering that some similarities could be found between interstitial lung disease developing after SARS-CoV-2 infection and in some autoimmunological diseases, it is recently hypothesized that immunosuppressive and biologic agents could play a therapeutic role in the treatment of COVID-19 patients [82]. Similarities between idiopathic pulmonary fibrosis (IPF) and pneumonitis in the course of COVID-19 are also observed; thus, antifibrotic drugs, as used in IPF treatment, are deemed to be used [83,84]. Some patients developing severe lung fibrosis after non-resolving COVID-19 were successfully treated with lung transplantation [85].

### 4.2. Pulmonary Embolism

One of the critical complications of COVID-19 is pulmonary embolism (PE). The biological mechanisms leading to PE in COVID-19 pneumonia are complex and may include increased proinflammatory cytokines, endothelial injury caused by SARS-CoV-2, and platelet activation [86]. A hypercoagulable state that develops during the course of COVID-19 patients predisposes to intravascular pulmonary thrombosis and PE [87].

Most hospitalized patients with COVID-19 do not have the traditional risk factors for PE [88,89]. In addition, PE can develop in patients with COVID-19 despite anticoagulation prophylaxis [78,79,80]. At the time of admission, patients with increased heart rate, impaired right ventricular function, elevated D-dimer and CRP levels, and decreased arterial oxygen saturation when breathing room air are at higher risk of developing PE [89,90]. The longer time that elapsed from initial symptoms to hospitalization (median 14 days versus 7 days) was associated with an increased risk of PE [91].

In hospitalized COVID-19 patients, PE was diagnosed on average about three weeks after the first symptoms [92]. PE may also occur several days after hospitalization in patients with moderately severe COVID-19 pneumonia [93]. In patients who required treatment in the intensive care unit, PE developed a median of six days after admission [94]. Massive PE can also occur in initially asymptomatic COVID-19 patients, as shown in a post-mortem study [95]. In most COVID-19 patients, PE was detected in peripheral rather than central pulmonary vessels [89,92]. Its localization corresponded in most cases (71%) to radiological abnormalities in the form of consolidations on chest radiographs [89]. Coexisting PE significantly increases the mortality rate in patients with COVID-19 pneumonia [86,96,97]. In the study of acute respiratory distress syndrome (ARDS) developing in COVID-19, PE was diagnosed five times more frequently than in non-COVID-19 ARDS patients [98]. Among patients treated in the ICU, the incidence of PE was approximately three times higher in COVID-19 patients than in patients with influenza [94]. PE incidence in COVID-19 patients treated in the ICU ranged from 11.7% to 20.6% [94,98,99] and in other hospitalized COVID-19 patients—from 2% to 17.6% [86,88,100,101,102]. However, when CT pulmonary angiography was performed in all patients, even without clinical suspicion of PE, the frequency of this diagnosis ranged from 14.2% [91] to 35.6% of patients [90]. PE may represent a clinical picture of remote sequelae of COVID-19 [103].

Low-molecular-weight heparin in prophylactic doses has been recommended to prevent PE in hospitalized patients with COVID-19. However, the need to increase usual doses of thromboprophylaxis in patients with COVID-19 has been postulated [100,104,105]. The exact dosage of thromboprophylaxis in patients with COVID-19 has not yet been clarified and has awakened the results of multicenter studies [94]. To support early antithrombotic treatment, it is recommended to monitor D-dimer levels, fibrinogen, prothrombin time, and platelet count [106]. Systemic thrombolysis is suggested for selected patients [102].

### 4.3. Pneumothorax

Pneumothorax can result from barotrauma in ventilated patients with the most severe form of COVID-19 lung involvement [107]. Pneumothorax may also develop spontaneously (secondary spontaneous pneumothorax), i.e., without known lung or chest injury, usually in the later phase of COVID-19 pneumonia with damaged alveolar wall [108]. Spontaneous pneumothorax developed during hospitalization at a median of day nine in a group of 22 patients out of 1690 hospitalized with COVID-19 [104]. The incidence of spontaneous pneumothorax has been reported to range from 1–1.4% [104,109,110] to 17.65% [111]. Treatment of pneumothorax may require the insertion of a chest drain. This severe complication may be a sign of poor prognosis [112] and has been associated with a risk of death of up to 36% [104].

Spontaneous pneumothorax may be a rare late complication of COVID-19 pneumonia that occurs in patients with an initially mild course of disease that does not require mechanical ventilation [113].

## 5. Long COVID Syndrome and Rehabilitation

Long COVID is a new term that describes a longer, more difficult course of disease than the initial signs of SARS-CoV-2 infection [114]. The persistence of symptoms or the development of new symptoms associated with COVID-19 presents a new medical challenge [106].

A surprising feature of post-COVID-19 syndrome is that it affects survivors of COVID-19 at all levels of disease severity [105]. A growing body of literature has shown that long COVID can also affect mild to moderate cases [115]. Of even greater concern, post- COVID-19 syndrome appears to affect children, even those who were asymptomatic COVID-19 [116,117].

Convalescents continue to suffer from a variety of symptoms for months. Without a clear clinical definition of the criteria for medical diagnosis of long COVID, the healthcare system will not meet the needs of patients [118]. Recovered individuals, also referred to as “COVID long-stayers”, have been classified as those who have symptoms more than 28 days after diagnosis [106]. Many studies have published that most patients experience at least one symptom during their recovery period [119,120]. Radiological abnormalities in the lungs associated with persistent symptoms are still present in approximately half of COVID-19 survivors six months after the onset of symptoms [121]. In a three-month follow-up study published by Zhao et al., pulmonary radiological abnormalities were found in 71% of COVID-19 survivors and functional impairment in 25% of participants, although only less than 10% had severe pneumonia [77]. Symptoms occurring during COVID-19 syndrome may be single, multiple, constant, transient, or fluctuating and may occur with varying frequency [106]. The most common persistent symptoms are fatigue, dyspnea, chest pain, joint pain, palpitations, anosmia and dysgeusia, hair loss, cognitive symptoms (memory and attention deficits), and psychosocial problems [121,122,123,124]. In the most comprehensive study of 1733 patients, Huang et al. found fatigue or muscle weakness in 63% of patients, sleep disturbances in 26%, and anxiety or depression in 23% of patients after a 6-month follow-up [121].

Post-COVID-19 syndromes can be expected to account for a large proportion of consultations in both primary and secondary care in the near future [125,126]. As the pandemic does not seem to be subsiding and the world is facing a new wave of COVID-19 [122], the major challenge for the healthcare system at present is not only to manage the acute phase of SARS-CoV-2 infection but also to plan rehabilitation strategies [123]. Early implementation of rehabilitation is crucial for patients with COVID-19 to effectively prevent further deterioration of the disease and reduce the risk of developing severe complications after COVID-19 [124]. Rehabilitation has been shown to improve respiratory function and awareness, shorten the time of mechanical ventilation, reduce the risk of complications, and decrease the length of hospital stay, risk of readmission, and mortality [125]. As reported above, discharged patients should clearly be offered long-term access to multidisciplinary healthcare, including rehabilitation services.

## 6. Continuing Mutation of SARS-CoV-2

Long COVID is a new term that describes Another very important aspect of SARS-CoV-2 is the continuing mutation of this virus [127,128,129]. At the beginning of the year 2022, the Omicron variant became the predominant cause of the disease [130]. Infection with the Omicron variant caused milder disease with much fewer need for hospitalization [131,132]. There were less frequent lower respiratory tract infections caused by the Omicron variant and thus less frequent severe forms of the disease were observed [133].

In addition, the probability of symptoms persisting beyond two months in the patients infected with the Omicron variant appeared to be diminished as compared with ancestral COVID-19 [134,135]. Long-COVID symptoms in the patients infected in the period of the Omicron variant predominance differed from the symptoms in the patients infected in the period of predominant Delta variant, with a higher incidence of cough (20% vs. 7%), fatigue and insomnia, and lower incidence of dysosmia and dysgeusia [131].

## 7. Conclusions

SARS-CoV-2 infection is currently the greatest public health challenge. As the COVID-19 pandemic subsides, the primary concern of the global health system appears to be shifting from treating all COVID-19 cases of acute respiratory distress syndrome to managing the short- and long-term complications of COVID-19. There are emerging data on a broad spectrum of cardiovascular, pulmonary, and neuropsychiatric sequelae following SARS-CoV-2 infection. Given the complexity of COVID-19, affected patients may require long-term follow-up care that is best provided by multidisciplinary teams. Numerous studies demonstrate that significant physical, psychological, and cognitive impairments may persist despite clinical resolution of infection and regardless of disease severity. It is therefore evident that early rehabilitation interventions for patients with SARS-CoV-2 infection are necessary to successfully prevent further deterioration of the disease and reduce the risk of developing severe, long-term complications.

## Figures and Tables

**Table 1:** Blood pressure thresholds and targets. A comparison between ACC/AHA, ACP/AAFP, and ESC/ESH guidelines.

	ACC/AHA 2017	ACP/AAFP 2017	ESC/ESH 2018
Definition of Older Patients	≥65 years	≥60 years	Elderly 65–79 years Very Old ≥ 80 years
BP Threshold for Initiation of Pharmacotherapy	≥130/80 mmHg	SBP ≥ 150 mmHg	Elderly ≥ 140/90 mmHg Very Old ≥ 160/90 mmHg
Blood Pressure Target	<130/80 mmHg	SBP < 150 mmHg	SBP 130–139 mmHg DBP 70–79 mmHg
